# Perovskite versus Standard Photodetectors

**DOI:** 10.3390/ma17164029

**Published:** 2024-08-13

**Authors:** Antoni Rogalski, Weida Hu, Fang Wang, Yang Wang, Piotr Martyniuk

**Affiliations:** 1 Institute of Applied Physics, Military University of Technology, 2 Kaliskiego St., 00-908 Warsaw, Poland; antoni.rogalski@wat.edu.pl; 2 State Key Laboratory of Infrared Physics, Shanghai Institute of Technical Physics, Chinese Academy of Sciences, 500 Yu Tian Road, Shanghai 200083, China; wdhu@mail.sitp.ac.cn (W.H.); yang_wang@fudan.edu.cn (Y.W.)

**Keywords:** fundamental limits of photodetector performance (SFL and BLIP), photogating effect, perovskite photodetectors, HOT devices, colloidal quantum dot (CQD) detectors

## Abstract

Perovskites have been largely implemented into optoelectronics as they provide several advantages such as long carrier diffusion length, high absorption coefficient, high carrier mobility, shallow defect levels and finally, high crystal quality. The brisk technological development of perovskite devices is connected to their relative simplicity, high-efficiency processing and low production cost. Significant improvement has been made in the detection performance and the photodetectors’ design, especially operating in the visible (VIS) and near-infrared (NIR) regions. This paper attempts to determine the importance of those devices in the broad group of standard VIS and NIR detectors. The paper evaluates the most important parameters of perovskite detectors, including current responsivity (*R*), detectivity (D*) and response time (*τ*), compared to the standard photodiodes (PDs) available on the commercial market. The conclusions presented in this work are based on an analysis of the reported data in the vast pieces of literature. A large discrepancy is observed in the demonstrated *R* and D*, which may be due to two reasons: immature device technology and erroneous D* estimates. The published performance at room temperature is even higher than that reported for typical detectors. The utmost D* for perovskite detectors is three to four orders of magnitude higher than commercially available VIS PDs. Some papers report a D* close to the physical limit defined by signal fluctuations and background radiation. However, it is likely that this performance is overestimated. Finally, the paper concludes with an attempt to determine the progress of perovskite optoelectronic devices in the future.

## 1. Introduction

The term “perovskite” was introduced when the mineral calcium titanate (CaTiO_3_) was discovered by Gustav Rose in 1839 in the Urals, and later named in honour of the mineralogist L.A. Perovski, who conducted extensive research on its structure. Since then, the term perovskite has been expanded to include all compounds exhibiting an identical or similar crystal structure to CaTiO_3_. Currently, perovskites are promising materials for future applications to include typical optoelectronic devices (photodetectors, light-emitting diodes (LEDs), lasers and solar cells (SCs)) [1,2], neuromorphic devices (cutting-edge technologies) like artificial synapses/memristors and finally pressure-induced emissions. That development was mainly activated by the progressive evolution of the solid-state perovskite, which is considered as a robust candidate for next-generation solar cells (SCs). The most efficient perovskite devices currently outperform industry-standard multicrystalline Si SCs, despite the fact that perovskites are normally grown at low temperatures using simple solution-based methods [3]. The wide use of perovskites beyond photovoltaics (PVs) is conditioned by the feasibility of tuning optoelectronic properties. The reported performance at room temperature is even higher than that presented for typical commercial photodetectors. Several papers report the detectivity (D*) being close to the limits of signal fluctuations and background radiation. It appears that the authors of these papers are not aware of those limitations. There are many papers that have reported on perovskite-based photodetectors, for this reason, this paper is limited to the most important parameters affecting their further development in the future. This paper highlights the unique perovskite properties and their effect on the photodetectors’ performance. It is also documented that the performance of the perovskite-based photodetector was found to be overestimated in many cases due to erroneous noise estimates, miscalculations of the device’s active area and light power density, and conflicting bandwidths adopted for the measured noise and sensitivity.

## 2. Fundamental Properties of Perovskite Materials

**Figure 1:**
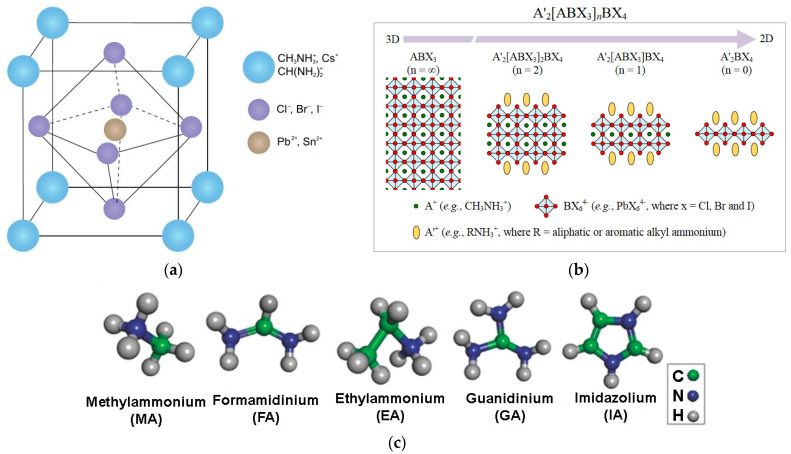
Perovskite structures: (**a**) the 3D ABX_3_, where A corresponds to the FA, MA, Cs^+^ cations, B denotes ${Sn}_{2}^{+}$ and ${Pb}_{2}^{+}$ metal cations and X stands for Br^−^, Cl^−^, I halogen anions (after Ref. [4]); (**b**) 2D (RNH_3_)_2_[ABX_3_]nBX_4_ (n = 0, 1, 2) and 3D ABX_3_ (n = ∞) perovskite structures, where A′ represents the ${RNH}_{3}^{+}$ cations (after Ref. [4]); (**c**) molecular structures of different organic amine cations.

Perovskites are characterized by a nearly cubic structure with a unit cell given in the formula of ABX_3_, where cation A may stabilize octahedra [BX_6_]^4−^ built by cation B and halide X (Cl^−^, Br^−^, I^−^) (see Figure 1). In terms of the photoelectrical conversion properties, the proper cations for metal halide perovskites are limited. Organic molecules, such as ${CH}_{3}{NH}_{3}^{+}$ (MA—methylammonium) and $CH{\left(CH_{2}\right)}_{2}^{+}$ (FA—formamidinium) and inorganic cations, such as Ru^+^ and Cs^+^, are used as monovalent A cations, while bivalent transition metal ions Mn^2+^, Pb^2+^, Sn^2+^, and Ge^2+^ are implemented as B cations.

By changing the constituent elements of the perovskites, the electrical and optical properties and the stability may be significantly changed. The crystal structure depends on the ionic radius of the A-site cations due to the restriction of the octahedral [BX_6_]^4−^ framework. To assess stability, Goldschmidt introduced both the tolerance (*t*) and the octahedral (*μ*) factors [5]. The $t=\left(r_{A}+r_{X}\right)/\sqrt{2}\left(r_{B}+r_{X}\right)$ allows for the assessment of the state of distortion and the octahedral factor can be calculated using $\mu =r_{A}/r_{X}$, where *r_A_*, *r_B_*, *r_X_* represent the A-, B-, and X-site cations ionic radii, respectively. Generally, ideal cubic, stable perovskite structures may be reached at 0.9 < *t* < 1.10, while tetragonal and orthorhombic structures may be formed at 0.81 < *t* < 0.9.

**Figure 2:**
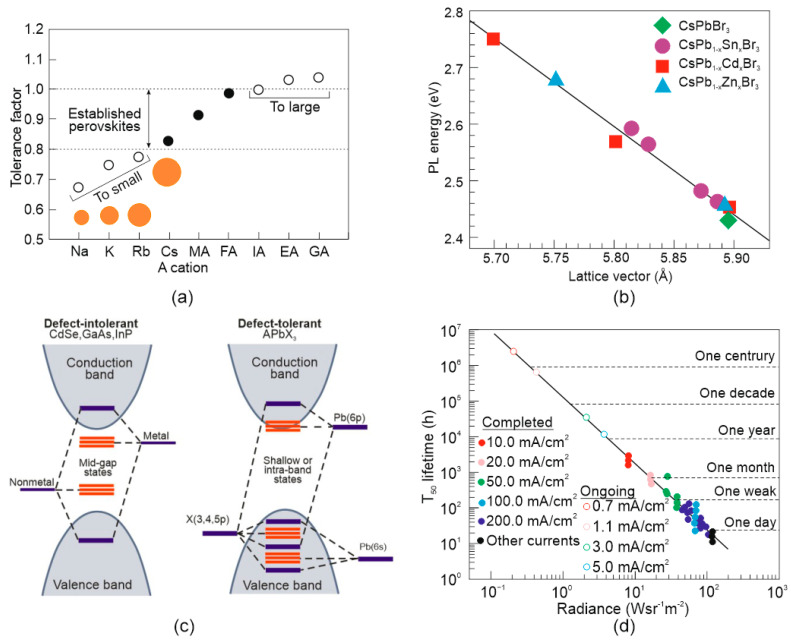
Properties of perovskite materials: (**a**) APbI_3_ tolerance factor for selected cations in the A-site [6]; (**b**) photoluminescence energy for doped CsPb_1−x_M_x_Br_3_ nanocrystals versus the lattice vector for M = Sn^2+^, Zn^2+^, Cd^2+^ [8]; (**c**) the band structure of typical defect-intolerant semiconductors (e.g., GaAs, CdSe, InP) (left) and defect-tolerant halide perovskite (e.g., APbX_3_) (right) [9]; (**d**) the T_50_ lifetimes versus initial radiance. The solid dots are based on the T_50_ measurements, while the open circles depict the extrapolated T_50_ lifetimes for the ongoing measurements at medium and low current densities [10].

Figure 2a presents the tolerance factors’ variation for the selected A-site cations, where HC(NH_2_)_2_^+^ (FA), Cs^+^, CH_3_NH_3_^+^ (MA) may yield stable perovskite materials, while any smaller (Na^+^, Rb^+^, Ka^+^) may distort the crystal structure [6]. On the other side, organic cations (e.g., ethylamine (EA^+^), guanidinium (GA^+^), imidazolium (IA^+^)) with larger ionic radii induce the growth of the in-plane direction octahedral [BX_6_]^4−^ and cannot enter the octahedral gap.

The perovskite energy bandgap depends on the A-site cations’ selection with different ionic radii in the octahedral space. This causes a contraction or expansion of the perovskite crystal lattice due to the tilt of the inorganic octahedral space and changes both the bond length and the B–X bond angle. Generally, increasing the size of the A-site causes a redshift in absorption. It turns out that A-site cations engineering composition provides a solution for producing perovskites with a wider range of tuneable energy gaps in the receptive band when the composition engineering of halides is ineffective.

Both A- and B-site cations determine the stability, where the selection of B-site cations should meet the Goldschmidt tolerance and octahedral factors. The standard 3D perovskites exhibit 0.442 ≤ μ ≤ 0.895 [7]. The B-site cations are positioned at the centre of the octahedron and determine both the crystalline phase by changing the rotation or octahedral inclination and regulating the emission properties and electron levels. The [BX_4_]^6^—octahedron orbit (the bond angle and band length of B-X) determines the energy band structure near the band edge, directly influencing the energy gap. For example, Figure 2b shows absorption and emission spectra blue shift for doped CsPbBr_3_ nanocrystals as linear versus the lattice contraction [8]. CsPbBr_3_ nanocrystals were doped using Zn^2+^, Cd^2+^, and Sn^2+^ through post-synthetic cation-exchange mechanisms. During this process, the Pb^2+^ cations are partly changed by the doped cations with a lower ionic radius, reducing the Pb–Br bond length.

Unlike typical semiconductors, where defect trap states are placed between the bottom of the conduction band (CB) and the top of the valence band (VB), the perovskite orbitals are positioned inside or near the VB and CB band edges (see Figure 2c) making perovskites highly tolerant to defects [9]. These defects do not act as trap states and do not influence the device’s electronic and optical performance. This benefit is particularly evident in flexible LEDs, which must withstand various mechanical deformations.

Thermal and structural instability is the critical problem that needs to be solved for Pb perovskite with organic cations [11]. The 3D MA-based perovskites were found to be unstable due to the fairly hazardous MA cations [12]. Moreover, MAPbI_3_ is susceptible to heat, moisture, oxygen and light causing material degradation while FA-based perovskites were assessed to be much more thermally stable than MA. Generally, inorganic perovskites (CsPbI_3_) exhibit higher thermal stability; however, the black phase with high power conversion efficiency (PCE) was not found to be the most stable. The published studies in Ref. [13] prove that mixed MA/FA perovskites exhibit better stability and performance. It appears that MA composition drives a desirable FA perovskite crystallization into its photoactive black phase, being both more thermally and structurally stable. Moreover, the spectral responsivity may be tuned within the range of 1.48 eV (FAPbI_3_) to 1.73 eV (CsPbI_3_) where the bandgap varies by the substitution of A cations. The bivalent Pb ion is generally the optimal selection for B cations to reach a better absorber region despite the Pb toxicity to the human body and environment. Several elements were implemented to replace Pb, among them Sn, Ge, Bi and Cu [4].

More recently, it was discovered that by using a dipolar molecular stabilizer (sulfobetaine 10 (SFB10)), it is possible to produce efficient and stable perovskite LEDs with much longer lifetimes to meet the requirements of commercial applications—see Figure 2d [10]. The stability is driven by the introduction of a dipolar molecular stabilizer that reacts with anions and cations at the perovskite grain boundaries. The stabilizer slows down the transport of ions in the electric field, thus blocking the creation of lead-iodide that facilitates the phase transformation and decomposition of α-FAPbI_3_ perovskite. This data significantly eliminates concerns about perovskite’s instability, opening a faster path to industrial applications and suggesting that perovskite devices are not “inherently defective” in terms of stability.

Following the perspective performance of the 2D materials, the 2D perovskite-based materials also have great potential for practical applications. In comparison with the 3D bulk crystals, the 2D perovskite reaches an equivalent performance exhibiting better humidity resistance subsequently influencing the long-term stability and higher luminescence efficiency.

Perovskites are fabricated using simple and flexible low-energy methods. The metal halide perovskite ionic feature provides a low-cost solution precursor method via “wet chemistry” being similar to organic semiconductors and colloidal quantum dots (CQD). Nearly all of the solution processing methods are implemented to fabricate perovskite films for optoelectronic devices including spray/spin/blade-coating and roll-to-roll printing; however, it is not trivial to have high-quality material without defects. Polycrystalline films are generally the best selection for high PCE perovskite SCs, while for LEDs, nanocrystals (NCs) and quantum dots (QDs) are more useful. In turn, for lasers, single crystals (SCRs) and nanowires (NWs) are more suitable as they require a higher crystal quality.

**Figure 3:**
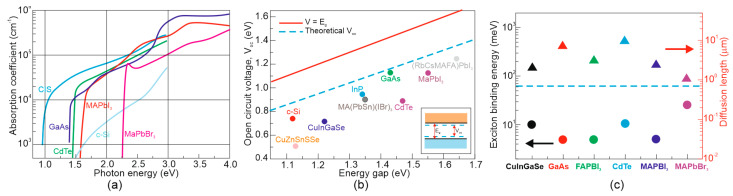
Optoelectronic material properties for the thin−film perovskite technologies: (**a**) absorption coefficient; (**b**) open−circuit voltage for the market technologies; and (**c**) binding energy and diffusion length for selected PV materials (after Ref. [15]).

The perovskite absorbers combine various benefits that contribute to the high photodetectors’ performance [13,14]. The significant optoelectronic properties (exciton binding energy, diffusion length, absorption coefficient and open circuit voltages of the perovskite SCs compared to other PV thin films) are presented in Figure 3 [15].

Table 1 summarizes the characteristic properties of the perovskite materials [16]. The high performance of the perovskite photodetectors is credited to their high electron mobility (up to 800 cm^2^/Vs) along with a long diffusion length (>1 μm). Absorption coefficients are reported at the level of 10^5^ cm^−1^ (considerably higher than for Si), caused by s-p antibonding coupling and a low exciton binding energy of <10 meV (significantly lower than 300 K thermal energy) allowing the generated carriers to be transported very rapidly as free carriers [17].

Single perovskites are the most advantageous among the crystal states and exhibit better electrical and optical parameters than microcrystals and polycrystalline films. This is related to their structural advantages such as free grain boundaries, an ordered long-range crystal structure and their high orientation, resulting in greater stability. Table 2 collects the basic material parameters such as carrier lifetime (*τ*), diffusion length (*L_D_*) and carrier mobility (*μ*) [18].

In comparison with polycrystalline perovskites, the SCR materials exhibit several advantages including better thermal/moisture stability, high purity and few grain boundaries. The better crystal quality drives low trap density, long carriers’ diffusion lengths, high carriers’ mobility, and fast/slow-component excitons lifetimes. The perovskite SCRs are synthesized using several methods: antisolvent vapour-assisted crystallization (VAC), top/bottom-seed solution growth (TSSG), solvent acidolysis crystallization and inverse temperature crystallization (ITC) being the simplest and most typically implemented methods [19].

For lead-iodide perovskites presently used for the highest PCE SCs, such as MAPbI_3_ and FAPbI_3_, both hole and electron mobilities were found to be primarily confined to the level of ~200 cm^2^/(V s), which is lower than for GaAs. Despite this, the MHPs exhibit high charge extraction and the net LD also depends on the recombination lifetimes being higher than standard GaAs [20].

CH_3_NH_3_PbI_3_ is the most frequently used and the constituent halide, metal and amine may be easily changed to build the different materials. It is feasible to modify the amine from MA ${(CH}_{3}{NH}_{3}^{+})$ to alternative amines. Another amine substitution may be the FA cation introduction ${(NH}_{2}{CH=NH}_{2}^{+}$) with the 1.47 eV bandgap energy being a more optimal SC energy gap than the 1.55 eV reached by MA-based perovskites [21]. As mentioned above, the completed mixed cation perovskites using the FA and MA cations are characterized by improved stability.

## 3. Performance Limitations of Photodetectors

Depending on the light interaction mechanism, the photodetector family is subdivided into further groups: intrinsic detectors, extrinsic detectors, photoemissive detectors (Schottky barriers) and quantum well detectors. The selected photodetectors are sketchily described in Ref. [22]. It can be concluded that they may be broadly split into two groups: photovoltaic (PV) and photoconductive (PC). Their distinctive parameters are summarised in Table 3.

To improve the responsivity of low dimensional solid (LDS) photodetectors (including 2D material-based detectors), FET/hybrid designs are often used; however, the majority of those devices exhibit a limited linear dynamic range (LDR) caused by the charge relaxation time saturating the available photoexcitation states, leading to a decrease in responsivity versus optical power (see the last line of Table 3). The applications require high-performance detectors exhibiting a wide LDR, meaning that the photocurrent exhibits a linear dependence on the incident radiation power before absorption saturation (*I_ph_* ∝ *P^α^*, where α is close to 1). In terms of the LDS detectors, the complicated carrier generation-recombination and trapping mechanisms drive exponent 0 < *α* < 1 determining the detector’s responsivity according to the equation *R* = *I_ph_*/P, so *R* ∝ *P*^−(1 − *α*)^. Both net photocurrent and responsivity are nonlinear functions of radiation power. A similar dependence is observed for photoelectric gain (*g*), which is strongly influenced by traps (see figure in the last line of Table 3). If radiation power increases, the carriers are progressively captured filling traps, which decreases carrier lifetime and photoelectric gain, while for low radiation power range, the sensitivity is not affected because of the high trap state density. Generally, the sensitivity being measured for different radiation powers is not used for comparing the detectors’ performance. A power density that is several orders of magnitude lower than 1 mW/cm^2^ is commonly used to estimate the current responsivity [23].

The photodetectors reach the most favourable conditions when the intrinsic detector noise is low in comparison to the photon noise [24,25,26]. The photon noise level is not related to the deficiencies in the detector design or integrated electronics but is connected with the detection mechanism, being determined by the discrete nature of the electromagnetic radiation field. The radiation incident on the detector consists of two parts, stemming from object to scene. There are two important factors fundamental to the detector’s performance: the signal fluctuation limit (SFL) and the background fluctuation limit, also known as the BLIP (background limited infrared photodetector). Both SFL and BLIP D* and their ultimate limits are given in Table 2.

**Figure 4:**
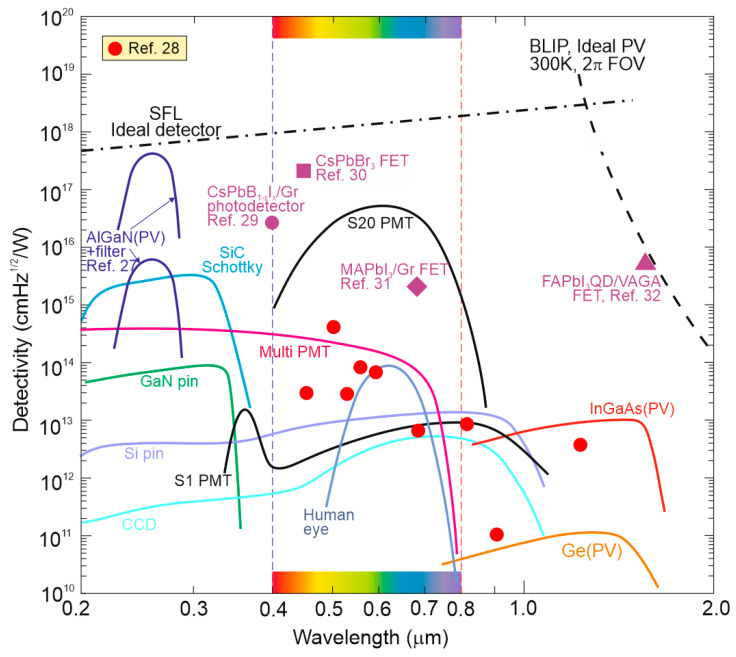
Comparison of room temperature D* for perovskite photodetectors [28,29,30,31,32] with standard market detectors (AlGaN, Si, Ge, InGaAs PDs and PMTs) in the wavelength range 0.2–2 μm. The ultimate SFL and BLIP are also shown. PV—photovoltaic detector, PMT—photomultiplier tube, FET—field effect transistor. The perovskite photodetectors’ D* marked in magenta is probably overestimated.

Figure 4 presents the ultimate D* reported for the selected photon detectors within a 0.2‒2 μm wavelength range with a 300 K background temperature and 2π field of view (FOV). As presented, the SFL and BLIP curves crossing is located at ~1.2 μm and <1.2 μm, where the device operates under SFL and the D* wavelength dependence is weak; while for >1.2 μm, BLIP dominates and D* dependence is strong resulting in an intense increase in the scene radiation influence at the edge of the spectral distribution at 300 K.

The experimental data highlighted in Figure 4 represent the standard photodetectors available on the commercial market and the rest of the data are limited only to high-performance perovskite photodetectors published in the literature. In terms of standard detectors, AlGaN photodiodes (PDs) exhibit the highest D* at 260 nm close to SFL. However, to reach that high D*, it is essential to use filters to suppress solar irradiance leakage [27]. The highest D* for perovskite detectors [28,29,30,31,32], marked in magenta, is located above those of standard photodetectors. In addition, the papers signaling the highest D* do not elaborate on the optical filters used during characterization. It is expected that the perovskite photodetectors’ D* marked in magenta is overestimated.

In order to estimate D*, the contribution of the different noises should be taken into account: shot, generation-recombination (gr), photon, thermal and 1/*f* noises. However, there are many reports that do not consider the photogain effect’s influence on the shot and gr noises mainly for room-temperature devices exhibiting low response time caused by long carrier lifetimes (typical for hybrid photodetectors—phototransistors (PTs)). Assuming improper equation for the shot noise ($I_{sh}=\sqrt{2qI∆f}$ rather than of $I_{sh}=\sqrt{2qgI∆f}$) causes false improvement (record-breaking performances) in the signal-to-noise ratio (SNR) by a factor of $\sqrt{g}$ in relation to the SNR at the detector input. A similar dependence on $\sqrt{g}$ occurs for gr noise since $I_{gr}=\sqrt{4qI_{d}g∆f/\left(1+\omega^{2}\tau^{2}\right)}$ (the gr noise is frequency dependent, ω). The shot/gr noises’ estimate error increases for higher *g* and is principally important for photodetectors exhibiting high internal gain. For further discussion on this topic see the next section.

There are several reports stressing the cases where the photodetector’s performance is overestimated by using the wrong characterization procedures, including inaccurate assessment of the following:

1. Noise;
2. The device’s active area and radiation power density;
3. The bandwidth of the measured responsivity and noise [33,34,35,36].

To eliminate D* overestimates, appropriate characterization procedures consistent with those used for typical standard photodetectors are required.

## 4. Design and Performance of Perovskite Photodetectors

**Figure 5:**
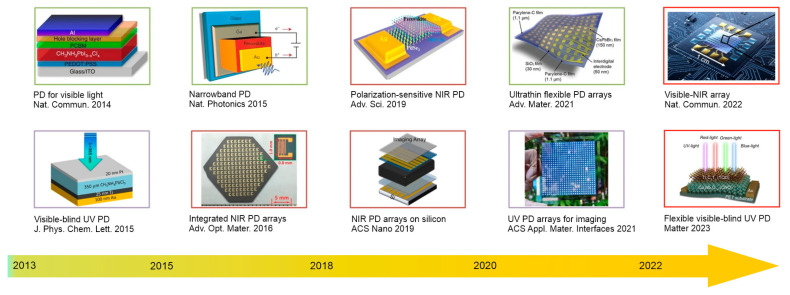
Timeline of perovskite UV–VIS–NIR photodetector development (adapted with Ref. [37] with the additions of VIS–NIR PD array and flexible VIS-blind UV PD).

Perovskite bandgaps may be varied through halide composition engineering for wide spectral band operation from X-ray to NIR. Perovskite photodetectors have been rapidly developing in recent years, as shown in Figure 5 for UV, VIS-blind and NIR photodetectors.

The perovskite-based detectors can operate in PC, PV and field effect transistor (FET) modes. The PC detectors are two-terminal devices (two ohmic metal–semiconductor contacts, or metal–semiconductor–metal (MSM) photodetectors) that allow photocurrent amplification. The PC effect results from the generation of extra free carriers by absorption of light in the semiconductor. The required polarization bias to balance photocurrent losses (capture of carriers in defects by non-radiative recombination) may be high for long distances between contacts in a planar configuration (from tens of μm to mm), while vertical PC configuration is characterized by a much lower distance between the electrodes (<0.5 μm).

**Figure 6:**
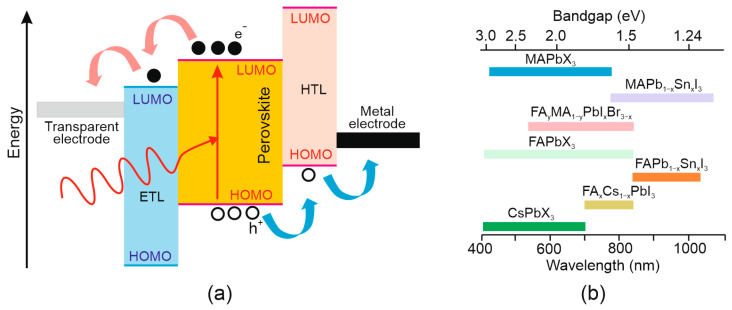
Perovskite PV detectors: (**a**) band diagram and principle operation (favourable energy band alignment affects the carriers’ transport to the device’s contacts); and (**b**) bandgap for perovskite materials.

The PV device, as is shown in Figure 6a, is built of three main regions, which are responsible for [21] photon absorption followed by carrier generation, carrier transport and finally carrier extraction. In the first region, exciton dissociation generates holes and electrons. The electron–hole pairs’ binding energies are significantly less than 300 K thermal energy, making the generation of the free charge carriers a very rapid process. Exciton dissociation occurs at the interface between the perovskite and the charge-transporting layer (CTL). Electrons are injected into the electron-transporting layer (ETL) and are transferred to the anode (fluorine-doped tin oxide—FTO glass). Concurrently, holes are injected into the hole-transporting layer (HTL) and next migrate to the metal cathode. As a consequence, the electrons/holes are gathered by contacts to generate current in the external circuit. For organic materials, the lowest unoccupied molecular orbital (LUMO) and highest occupied molecular orbital energies (HOMO) represent the lower and higher energy levels, respectively.

**Figure 7:**
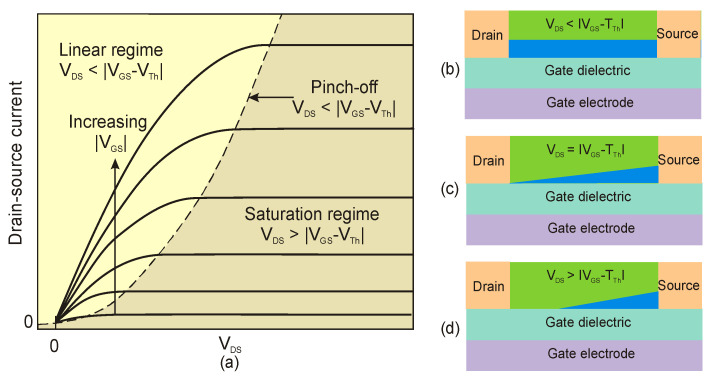
MOSFET operation: (**a**) output source-drain current curves for selected gate biases; (**b**–**d**) variation of the conduction channel versus *V_DS_*; (**b**) at linear; (**c**) at pinch-off; (**d**) in the saturation regimes.

Another important perovskite-based detector structure is the metal-oxide-semiconductor (MOS) FET, where two operating biases, the gate-source (*V_GS_*) and the drain-source (*V_DS_*), drive the operation. For *V_GS_* = 0, the device is “off” (if the flat-band condition is met; on the contrary, the threshold voltage V_th_ is needed to fill the charge traps at the semiconductor–dielectric interface). The applied (negative for a p-channel transistor) *V_GS_* leads to the accumulation of carriers at the semiconductor–dielectric interface. When the device is “on”, the *V_DS_* drives the drain-source current, as presented in Figure 7a,b. The majority of the carrier concentration in the p-channel of the transistor and the drain-source current will be driven by the electric field generated by the gate source, *V_GS_*. When the *V_DS_* reaches |*V_GS_* − *V_Th_*|, the region near the drain electrode is pinched off (Figure 7c), while the drain-source current saturates (Figure 7a) versus a further increase in *V_DS_* (Figure 7d).

The photogating mechanism is a specific example of the PC effect and can be realized in two ways:

1. Electron–hole pairs generation when one type of carrier is trapped by the localized states (nano-particles and defects);
2. Electron–hole pair generation in trap states where one type of carrier is transferred to nanostructured materials (generally LDS), whereas the other resides at the same place to modulate the layered materials.

The long carrier lifetime improves sensitivity but reduces response time. The trapped carriers in the localized states [see Figure 7a], creates local gate efficiently modulating the resistance of active materials. The photocarriers dynamics is driven by the localized trap states recombination lifetime determining the large photoconductive gain (*g*). The trap states, where carriers may be locked for long times are usually located at defects or at the surface of the active material. The trap states generation is of particular importance for nanostructured materials (LDSs) to include CQDs, NWs and 2D semiconductors, where the large surface and reduced screening determines the electrical properties. The photoelectric *g* = 1 is for typical PDs caused by the separation of minority carriers by the electrical field in the depletion region. For the hybrid LDS detectors, photosensing and carrier transport occurs in separately optimized areas: for effective light absorption and for providing fast charge reticulation what allows to reach ultra-high gain up to 10^9^ electrons per photon, and exceptional *R* for short wavelength IR (SWIR) photodetectors [2,3].

**Figure 8:**
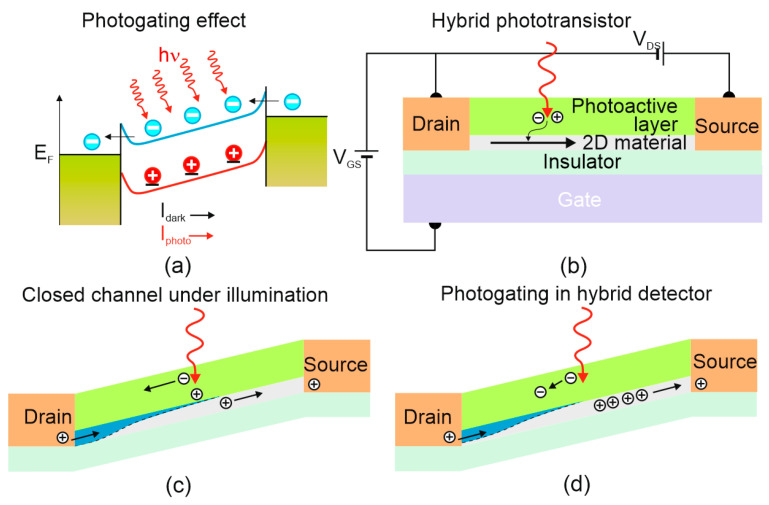
Photogating effect: (**a**) energy band structure under light condition (holes are trapped at the band edge acting as a local gate, while the field-effect drives more electrons in the channel, generating a photocurrent; if the electron lifetime exceeds the time it takes for the electron to transit device, then the trapped holes time allows the electrons to circulate resulting in high gain, (**b**–**d**) photogating effect in LDS based photodetectors; (**b**) the hybrid PT operation, (**c**) closed channel under light conditions, and (**d**) photoconductive gain.

The basic hybrid LDSs PT with the fast transfer channel for charge carriers is shown in Figure 8b. The graphene is not responsible for light absorption but only the charge sensing what is allowed by the graphene’s high ambipolar mobility (~10^3^–10^5^ cm^2^/Vs) acting as a built-in photogain (i.e., amplifier) effect increasing the detector response. The absorber’s selection determines the spectral response.

The atomic layer LDS is more vulnerable to the local electric fields than typical bulk materials and the photogating mechanism may effectively modulate the channel conductivity by external gate voltage, *V_GS_*. The optical gain enhancement is principally important due to the fact that the QE is constrained by the weak 2D materials absorption. For the hybrid detector presented in Figure 8d, the holes are introduced into transporting channel, while the electrons occupy the absorber layer. The injected charges may reticulate even several thousand times before recombination increasing gain under illumination. The carrier lifetime may be increased by both the bandgap and defect states engineering, while the trapping process limit the response time even to several seconds making a trade-off between optimized sensitivity and photoresponse time.

The perovskite photodetectors are fabricated with numerous morphologies to include SCRs, polycrystalline films (PFs), QDs/NCs, NWs/nanorods (NRs) and 2D/quasi-2D perovskites [11,19,37,38]. In comparison with PF perovskites, the SCRs materials have several advantages to include: better thermal/moisture stability, high purity and few grain boundaries. The better crystal quality drives low trap density, long carriers’ diffusion lengths, high carriers’ mobility, fast/slow-component excitons lifetimes. Perovskite SCRs are synthesized by several methods: antisolvent vapour-assisted crystallization (VAC), top/bottom-seed solution growth (TSSG), solvent acidolysis crystallization and inverse temperature crystallization (ITC) being the simplest and most typically implemented method [8]. Table 4 collects the perovskite photodetectors’ performance comparison [11,29,32,39,40,41,42,43,44,45,46,47,48].

**Figure 9:**
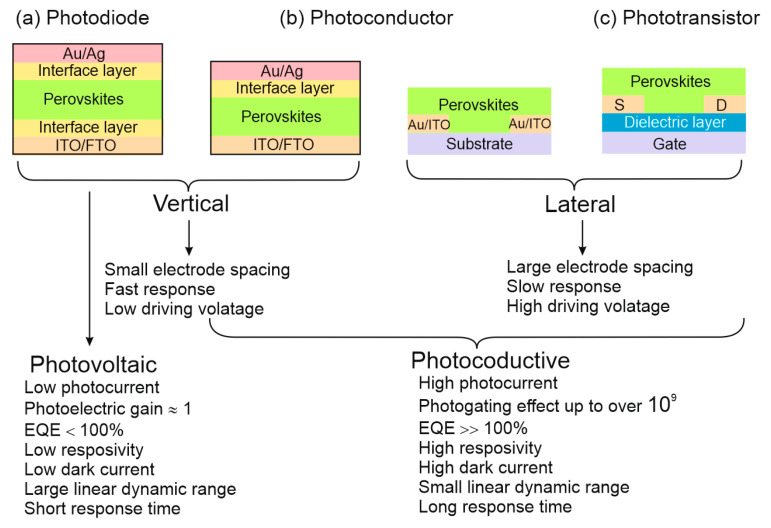
Pros and cons of the perovskite based photodetectors: PV, PC and PT.

Figure 9 schematically illustrates the characteristics of perovskite based photodetectors. Depending on the spatial arrangement of the active area and electrodes, perovskite photodetectors can be divided into vertical and lateral designs. In general, vertical detectors provide a low supply voltage due to the small electrode spacing and short carrier transit length. Lateral photodetectors, on the other hand, exhibit slow response and high driving voltage due to large electrode spacing. For PDs, the p-n junction determines both the low dark current and high detectivity. However, PDs suffer from low responsivity and external QE (EQE < 100%) because the photoelectric gain is close to 1. As mentioned earlier, both PC photodetectors and FET PTs show high *R*, EQE (well above 100%) and high gain. For PTs, the record published gain is as high than 10^9^ (for PC > 10^5^). However, high gain, in turn, usually results in a low response speed, because both response time and gain are determined by the lifetime of the carriers. Therefore, there are always inherent contradictions between responsivity and response speed. Table 4 collects the perovskite photodetectors performance comparison. In the following discussion, examples of perovskite based photodetectors design and performance are provided.

Table 4 collects the perovskite-based detectors’ performance comparison. In the following discussion, examples of perovskite-based detectors’ design and performance are provided.

**Figure 10:**
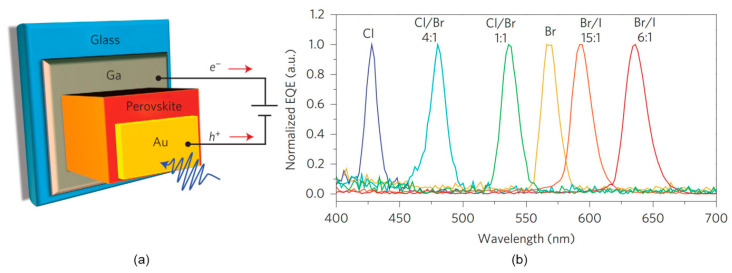
The mixed single crystal MAPbBr_3−x_Cl_x_ and MAPbI_3−x_Br_x_ narrowband perovskite detectors (**a**) device design, (**b**) normalized EQE versus selected halide compositions [measured for −1 V (after Ref. [49])].

Fang et al. adjusted the perovskite single-crystal halogen composition and fabricated narrowband photodetectors exhibiting a full width at half maximum (FWHM) < 20 nm [49]. The spectral response of the mixed MAPbBr_3−x_Cl_x_ and MAPbI_3−x_Br_x_ photodetectors may be adjusted between blue and red wavelengths. As is shown in Figure 10, the ~1 mm thick detector is built of the perovskite single crystal, semi-transparent Au anode and Ga cathode.

The selective detection process involves the carriers generated by the long wavelengths (long optical penetration depth) being collected typically in a thin region next to the Au electrode, while carriers excited by shorter wavelengths recombine before being transported to the electrode (strong surface-charge recombination). The optical penetration depth was reported <300 nm for the perovskites. That approach provides a new method to adjust the photodetector’s spectral responsivity without the need for optical filters.

Another strategy to develop wavelength-selective detection is charge collection narrowing [50]. The short wavelengths are primarily absorbed on the device’ surface (surface generation, high absorption coefficient) being the transparent conductive electrode. In turn, the long wavelength radiation penetrates much deeper (bulk generation) into the detector. In addition, the carriers’ collection and transport may be monitored by the absorber thickness and perovskite’s composition.

**Figure 11:**
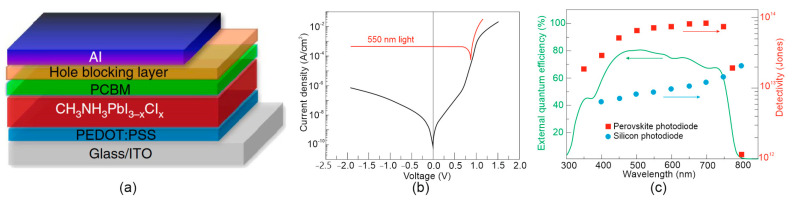
Typical perovskite PD: (**a**) heterojunction device design—structure incorporates ITO transparent conductive (ohmic) electrode, HTL (PEDOT:PSS), absorber (MAPbI_3 − x_Cl_x_), ETL (PCBM), and Al ohmic contact, (**b**) dark *J*-*V* and under illumination, (**c**) EQE and spectral detectivity with Si PD for comparison (after Ref. [51]).

Compared to Si PDs, the perovskite PDs exhibited exceptional performance: very low dark current/noise, fast response, high linear LDR, and high *R*/D*. The vertical PV photodetectors being the most popular designs, do not differ significantly from SCs architectures. An example of design of perovskite-based PD is shown in Figure 11a. As is shown, on top of ITO-coated glass, an organic–inorganic hybrid CH_3_NH_3_PbI_3–x_Cl_x_ layer is inserted between PEDOT:PSS (p-type HTL) and PCBM (n-type ETL). This typical perovskite PD structure incorporates transparent conductive (ohmic) electrode (ITO), HTL (PEDOT:PSS), absorber (MAPbI_3−x_Cl_x_), ETL (PCBM), and Al ohmic contact [51]. In the one of the first papers describing a perovskite PD, reported in 2014, D* ~ 10^14^ cmHz^1/2^/W (500–750 nm) for −0.1 V, rise *(τ_rise_*)/decay(*τ_decay_*) times of 180 ns/160 ns and LDR = 100 dB were obtained. To date, no major progress was reported, as can be deduced by comparing those values with the performance shown in Table 4.

**Figure 12:**
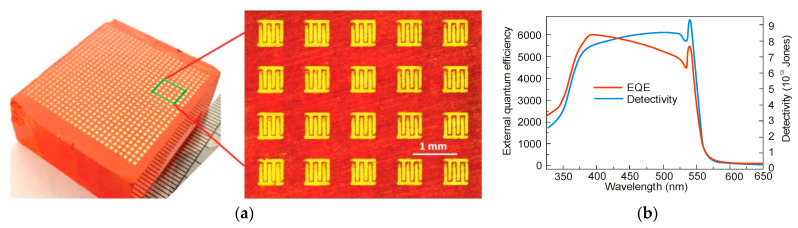
The 34 × 38 mm^2^ MAPbBr_3_ SCR based 27 × 27 array: (**a**) sensors’ picture, (**b**) PC’s EQE and D* versus wavelength for 4 V (after Ref. [52]).

Remarkable improvement has also been made in the perovskite photoconductors’ progress. Liu et al. presented ~1300 mm^2^ a large-area sensor built of 729 pixel array (Figure 12a) [52]. The PC array fabricated by MAPbBr_3_ SCR reaches high EQE, fast response time (40 μs) and high D* ~ 10^14^ Jones (Figure 12b)—much better than commercial sensors built of GaAs and Si. The performance of high-quality perovskite photoconductors is given in Table 4.

The FET are being considered as the third popular type of perovskite based devices. Typically, the high sensitivity is caused by the detectors’ low response rate related to the electrons trapped in possible defects at the interface between the dielectric and semiconductor. In addition to photoconductive gain, the electrical gating of the transistor introduces changes in the semiconductor Fermi level allowing to reach the high current sensitivity.

**Figure 13:**
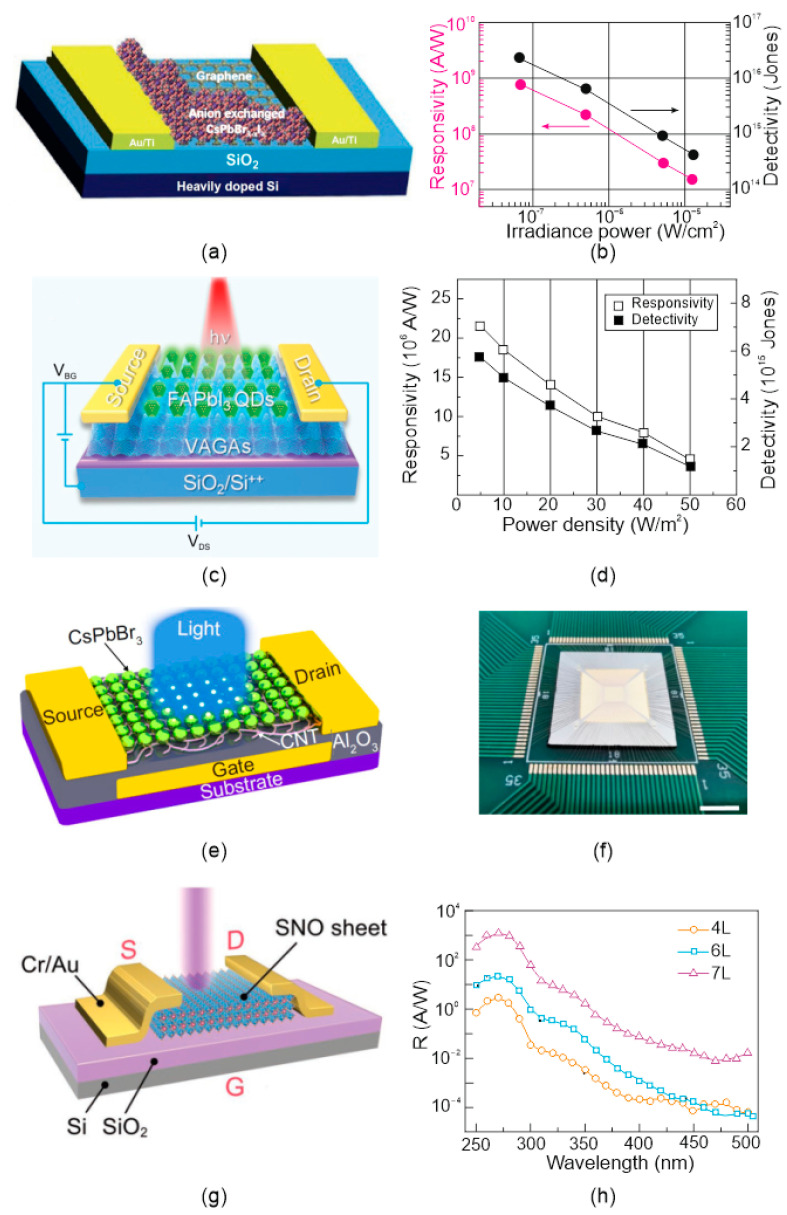
Perovskite PTs: (**a**) hybrid design based on graphene-CsPbBr_3 − x_I_x_ NCs and (**b**) *R* and D* for selected light powers for 405 nm and *V_DS_* = 1 V and *V_GS_ =* −60 V (after Ref. [29]); (**c**) FET NIR photodetector (*λ* = 1550 nm) based on FAPbI_3_ QD/VAGA and (**d**) power density-dependent *R* and D* (after Ref. [32]); (**e**) the PT design with a CNT/CsPbBr_3_-QD channel and (**f**) array with wire bonding on a printed circuit board (5 mm scale bar) (after Ref. [43]); (**g**) PT based on Sr_2_Nb_3_O_10_ (SNO) NSs and (**h**) the *R* for selected thicknesses for 1 V (after Ref. [54]).

Examples of perovskite FET PTs structures with different material configurations are shown in Figure 13. Generally, characteristics are similar for those observed for 2D FET PTs [53]. For the first detector structure shown in Figure 12a, CsPbBr_3 − x_I_x_ NCs stand highly photosensitive absorption region, instead the graphene employs a transport layer (amplifier caused by fast carrier transport in the graphene). This hybrid photodetector reaches *R* ~ 8.2 × 10^8^ A/W and D* ~ 10^16^ Jones for 0.07 μW/cm^2^ light power (405 nm) [29]. The slow rise and decay times (within few seconds) remain the severe bottleneck in photodetector application.

Another type of high performance vertical hybrid photodetector built of Gr arrays (VAGAs) and FAPbI_3_ QDs was reported by Feng et al.—see Figure 13c,d [32]. Modification of the FAPbI_3_ QDs by VAGAs increases the built-in potential in the hybrid structures. That improves the separation and the lifetime of photoexcited carriers. As presented in Figure 13d, the both *R* and D* decrease when the power density is reduced within the range of 50–5 mW/cm^2^. This effect is a consequence of the reduced LDR conditioned by the carrier relaxation time. The fabricated device shows high performance with D* ~ 5.64 × 10^15^ Jones and a high *R* ~ 2.17 × 10^7^ A/W @ 1550 nm. The light response rate with rise/decay time is in the miliseconds level (40 ms/46 ms).

Owing to the reduced cost, light weight, solution processing, wearability/bendability, the perovskite based photodetectors are widely implemented into electronic skins, stretchable displays and wearable devices. The flexible substrate selection depends on the device design. For backside illumination detectors the transparent substrates are required. Table 5 presents the key polymer parameters implemented into flexible photodetectors.

In recently published review, Zhang et al. presented the latest developments in perovskite based flexible photodetectors, including the device configurations (PC, PV, PT), fabrication methods (solution/vapour-based) and performance parameters [55]. As an example, we will present here the results of the paper published by Leung et al. [56].

**Figure 14:**
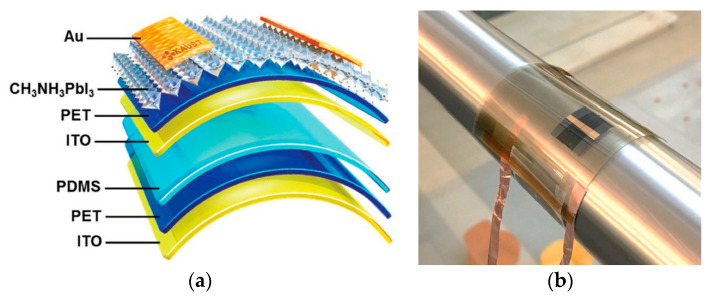
The flexible/self-powered perovskite based detector: (**a**) design, (**b**) device on the rounded surface (after Ref. [56]).

Figure 14a shows a design of the flexible and self-powered photodetector based on the perovskite methylammonium lead iodide (CH_3_NH_3_PBI_3_) fabricated by solvent engineering. This device may operate with random motion to include human finger tapping enabling operation without a massive external power source. The photodetector exhibits an remarkable D* ~ 1.22 × 10^13^ Jones and high *R* ~ 79.4 V/Wcm^2^. Moreover, it can operate under different bending angles [see Figure 14b]. In this way, the signal value exhibits a slight variation on light incidence angles indicating omnidirectional detection capability.

The perovskite photodetectors are also of interest for future utilizations in the human body and wearable technology including stimulated by the human eye—neuromorphic vision systems. An example of such a complementary structure is depicted in the Figure 13e [43]. This design is fabricated on flexible substrate with a metal gate and contains CsPbBr_3_ QDs atop a layer of semiconductor carbon nanotubes (s-CNTs). This heterostructure-based PT reaches a both record *R* ~ 5.1 × 10^7^ A/W and D* ~ 2 × 10^16^ cmHz^1/2^/W for 405 nm, *V_DS_*/*V_GS_* = 1/5 with response time in milliseconds. In addition, Ref. [43] demonstrates a 32 × 32 sensor array that can be used in a neuromorphic vision system—see Figure 13f.

The discovery of the 2D perovskite materials allowed to develop the next-generation optoelectronic devices to be split into two groups: non-/and van der Waals (vdW) types. The very first one stems from a 3D perovskite reduced to a single/few unit cells to include: nanoflakes (NFs), nanoplates (NPs) and nanosheets (NSs). The second group is processed by introducing organic long-chain cations into “A” place to prevent from the interaction of inorganic [BX_6_]^4−^ bilayers, primarily incorporating quasi-2D perovskites (RNH_3_)_2_[ABX_3_]nBX_4_ (n = 0, 1, 2, …). The improved humidity resistance, in comparison to the 3D perovskites, allowed 2D and quasi-2D perovskites to gain interest within the photo-electric-conversion field. Sheng et al. reviewed different methods of the quasi-2D perovskite crystals’ fabrication and their applications: LEDs, lasers, photodetectors and SCs [57]. The quasi-2D perovskite-based photodetectors were found to be mainly limited by the carrier’s transport issues attributed to the hybrid low-dimensional structure.

The 2D all-inorganic perovskite detector design based on the Sr_2_Nb_3_O_10_ (SNO) ~1.8 nm-thick NSs (processed by liquid exfoliation) is presented in Figure 13g [54]. That detector exhibits excellent UV@270 nm performance: narrowband *R* = 1214 A/W, EQE = 5.6 × 10^5^%, D* ~ 1.4 × 10^14^ cmHz^1/2^/W, 1 V, *τ_rise_* ≈ 0.4 ms, *τ_decay_* ~ 40 ms, surpassing the most 2D based UV detectors.

The perovskite photodetectors’ performance may be improved by bandgap engineering and hybridization with other materials. Ma et al. presented a PC heterostructure made of perovskite films [methylammonium lead triiodide (CH_3_NH_3_PbI_3_] and WS_2_ monolayers [58]. The WS_2_ layer was deposited on sapphire substrate by chemical vapour deposition (CVD) method, while perovskite layer by a thermal evaporation technique (TE). That allowed to reach high *R* ~ 17 A/W and D* ~ 10^12^ cmHz^1/2^/W. The WS_2_ monolayer carriers’ mobility and efficient interfacial charge separation allowed to increase the response time. Also the amorphous IGZO (indium gallium zinc oxide) exhibiting ~3 eV bandgap, high carrier mobility and low-temperature processing, was found to be flexible channel for hybrid organic–inorganic PTs. Du et al. fabricated and presented a hybrid phototransistor where solution-processed perovskite layer was deposited on IGZO [59]. A hybrid phototransistor sensitive to UV and VIS was fabricated with solution-processed organolead trihalide perovskite (MAPbI_3_) and deposited on the IGZO.

## 5. Performance Analysis of Perovskite Photodetectors

**Figure 15:**
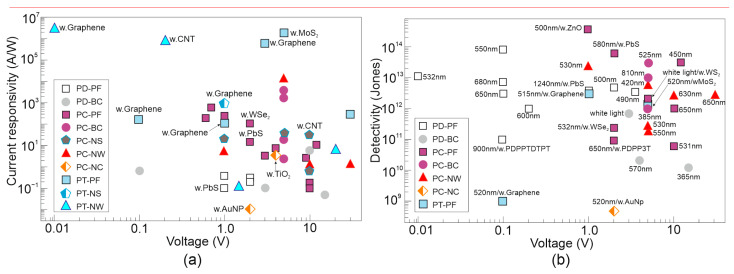
Detectivity (**a**) and current responsivity (**b**) versus voltage for device’ designs based on the perovskite materials published up to 2017. “w.Graphene” and similar (w.CNT, w.PbS, etc.) denote hybrid photodetectors with other materials. PD—photodiode; PC—photoconductor; PT—phototransistor; PF—polycrystalline film; BC—bulk crystal; NS—nanosheet; NW—nanowire; NC—nanocrystal (after Ref. [28]).

Figure 15 presents perovskite based photodetectors’ performance (*R* and D*) for selected material compositions, morphologies and device configurations published up to 2017 [28]. In general, comparing to PC, the PDs require a fairly low operating bias to reach a high D*. Higher current responsivities are presented for hybrid photodetectors (PTs) and photoconductors due to the influence of the photogating effect. The best perovskite photodetectors reach *R* ~ 10^5^ A/W and D* ~ 10^14^ Jones. These parameters indicate on the strong competitor for Si-based devices in sensing and imaging applications.

Over the past six years, many papers have been published demonstrating even better photodetector performance. In the following analysis, we will try to sort out the published performance of photodetectors and provide an analysis explaining the large discrepancies in their values.

**Figure 16:**
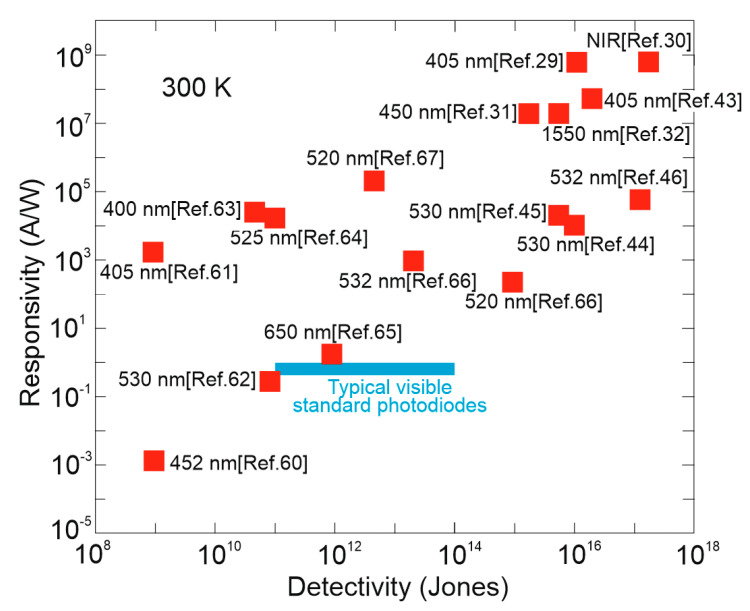
Comparison of *R* and D* for hybrid perovskite based photodetectors at 300 K. For comparison purposes, the typical VIS PDs performance is also marked [29,30,31,32,43,44,45,46,60,61,62,63,64,65,66,67,68].

At first, to benchmark presented results, Figure 16 collects the performance (*R* and D*) of selected perovskite based hybrid detectors (FET PTs) and compares with standard VIS PDs. The utmost D* for perovskite detectors are 3‒4 orders of magnitude higher than market crystalline Si PDs. Large discrepancy is observed in the demonstrated current sensitivity and detectivity, which may be due to two reasons: immature device technology and erroneous detectivity estimates.

**Figure 17:**
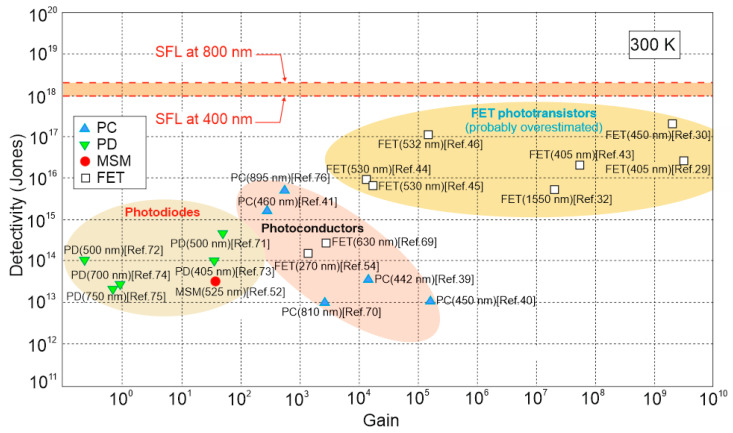
Detectivity dependence on gain for selected perovskite based detectors at 300 K. The are is collected after the following Refs. [29,30,32,39,40,41,43,44,45,46,52,54,69,70,71,72,73,74,75,76]. Theoretical predictions for SFL for 400‒800 nm wavelength range are also marked. PC—photoconductor, PD—photodiode, FET—field effect transistor, MSM—metal–semiconductor–metal.

Further insight on the detectors performance is shown in Figure 17 where the dependence of D* on gain for high quality perovskite based photodetector’ structures is presented. This figure also highlights theoretical predictions for SFL boundaries for 400‒800 nm wavelength range. The dominant areas of D*/*g* for each type of photodetectors—PDs, PCs and FET PTs—are also marked. The utmost D*, including partially overestimated (close to SFL), are marked for FET PT with large photogaiting effect (up to above 10^9^).

It is projected that the published record performance for perovskite based photodetectors is related to the device’s parameter overestimate. Section 3 of this paper discusses the causes of LDS performance overestimation due to improper noise estimation, inaccuracy of radiation power density and device’s active area, and assumption of different bandwidth of sensitivity and noise. It is expected that the main reason of the D* overestimation (close to the SFL) is improper/(or lack) of assumption of the internal gain in the gr/shot noises. The data from Ref. [32] and highlighted in Figure 4, Figure 15 and Figure 16, is for the NIR FET/FAPbI_3_QD/VAGA perovskite photodetector operating at 1550 nm and is close to the BLIP limit.

**Figure 18:**
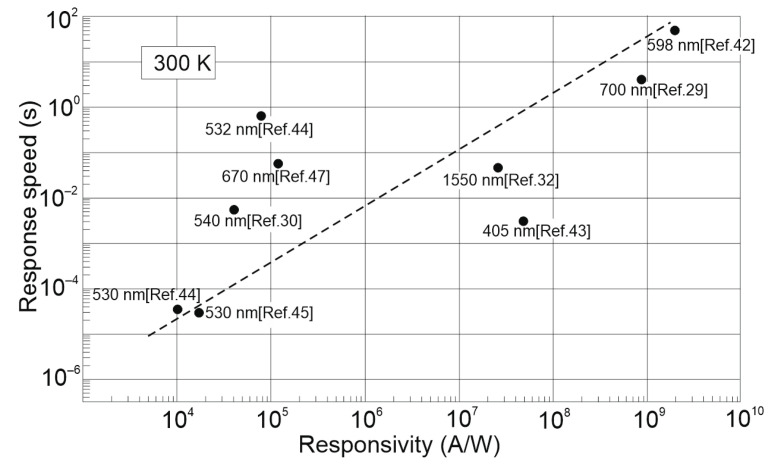
The room temperature current responsivity dependence on the response time for the perovskite-based hybrid photodetectors. The measured data were extracted from selected papers.

The gain influence on the photodetectors’ performance (shot/gr noises, responsivity) is explained in Table 3 and clarified in Section 3. The perovskite-based photodetectors, primarily hybrid (FET) devices, allow for an increase in responsivity; however, most of these detectors exhibit a suppressed LDR caused by the carrier’s relaxation time saturating the open photoexcitation states, leading to responsivity decreases versus light power. This effect is confirmed by the experimental characteristics shown in Figure 13b,d. The significance of that effect is the fact that sensitivity-optimized detectors exhibit a low response time, observed in the wavelengths and proved by the measured data shown in Figure 18. The time response up to a few seconds was reported for detectors with g > 10^9^ indicating the trade-off between photoresponsivity and response time.

## 6. Conclusions

The perovskite has developed as a perspective materials in last decade what is directly related to the excellent processability and improved carrier transport capabilities similar to the typical semiconductors allowing them to be easily implemented into numerous device’s applications to include: thin films SCs, LEDs, lasers, photodetectors, transistors, etc.

Below the most important capabilities of perovskites are articulated:

1. A wide variety of perovskites have a direct and tuneable energy gap controlled by mixing. Their electronic properties (doping) can also be controlled by the composition. Furthermore, the fabrication methods are relatively simple compared to organic materials (solution-based techniques at low temperatures lead to reasonable energy payback time); however, their physical properties are affected by other factors; e.g., crystallinity, morphology, grain size and processing history.
2. The pristine perovskites’ conductivity is relatively low; however, low effective mass allows the carrier mobility to be higher (fewer orders of magnitude) than organic semiconductors. The ultimate mobility limit is driven by the Fröhlich effect, and the carrier’s transport is influenced by the interaction with the electric field of LO phonons.
3. Perovskites’ development is mainly triggered by the progressive development of the solid-state perovskite next-generation SCs. Up to now, however, most of the perovskite materials and devices are still fabricated on the laboratory scale.
4. The perovskite-based detector’s performance was found to be overestimated in many cases due to: erroneous noise estimates, miscalculations of the device’s active area/light power density, and conflicting bandwidths adopted for the measured noise and sensitivity.
5. Several methods including carriers’ trap layers and photogating effect with fast channels may be introduced to increase sensitivity, but the carrier mobility and response time limitations restrict applications (trade-off between response time and sensitivity).

In terms of application, several issues remain unresolved:

1. Generally, the ionic nature perovskites are not that stable as inorganics. In a humid environment, they can drastically change the crystalline structure and composition causing permanent material damage. Effective housing is needed to fully protect the device while long-term stability under extremely harsh operating conditions also remains an issue.
2. Toxicity is another issue. So far, lead-based perovskites are the materials that exhibit the best performance; however, lead-free perovskites are gradually being introduced, e.g., bismuth-based and double perovskites.
3. another problem is miniaturization, particularly important in imaging arrays. The perovskite synthetization methods are completely different from silicon technology (e.g., conventional lithography, etching techniques) and a long term is necessary to develop the methods comparable with silicon. This question also concerns ROICs of perovskite arrays, where specific design is required (e.g., taking into account large RC sensor times).

To summarize the results analyzed and discussed in this paper, the perovskite-based devices exhibit the potential in optoelectronics especially in terms of abundance of constituent materials, excellent (compositional) flexibility and low-cost processing providing a distinct advantage over III-V materials. The biggest drawback of perovskites appears to be their instability (fragility, sensitivity to heat, humidity, oxygen, UV radiation). Linked to their nature, intrinsic electrochemical phenomena can damage them. If possible, a comment on the reliability o. However, recently published results suggest that perovskite devices are not “genetically defective” in terms of stability [10]. Further expansion of the perovskite materials family into optoelectronic applications is perceived to be still ahead.

## Figures and Tables

**Table 1:** Typical electronic and optical parameters for the perovskite materials (after Ref. [16]).

Parameter	Value
Energy gap	1.5–2.5 eV
Absorption coefficient	10^5^ cm^−1^
Exciton binding energy	<10 meV
Crystallization energy barrier	56.6–97.3 kJ/mol
Charge carrier lifetime	Greater than 300 ns
PL quantum efficiency (QE)	70%
Carrier mobility	800 cm^2^/Vs
Relative permittivity	3
Exciton	Wannier type exciton
Trap-state density	10^10^ cm^−3^ (single crystals) 10^15^–10^17^ cm^−3^ (polycrystals)

**Table 2:** Parameters of perovskite single crystals (after Ref. [18]).

Materials	Mobility (cm^2^/Vs)	Carrier Lifetime (ns)	Diffusion Length (mm)	Trap Density (cm^–3^)	Carrier Concen (cm^−3^)
MAPbCl_3_ (ITC) ^(1)^	For hole: 42 ± 9 (SCLC) ^(4)^	*τ_s_* = 83 *τ_b_* = 662 (TA) ^(6)^	3.0–8.5	For hole: 3.1 × 10^10^ (SCLC)	4 × 10^9^
MAPbBr_3_ (ITC)	For hole: 60 (SCLC and Hall effect)	242	A few micrometers	For hole: 1.6 × 10^11^ (SCLC)	For hole: 10^11^ (Hall effect)
MAPbBr_3_ (AVC) ^(2)^	115 (ToF) ^(5)^ 20–60 (Hall effect) 38 ± 5 (SCLC)	*τ_s_* = 74 ± 5 *τ_b_* = 978 ± 22 (TA) *τ_s_* = 41 ± 2 *τ_b_* = 357 ± 11 (TRPL) ^(7)^	3–17	(5.80 ± 0.6) *×* 10^9^ (SCLC)	For hole: 5 × 10^9^ 5 *×* 10^10^ (Hall effect)
MAPbI_3_ (ITC)	For hole: 67.2 *±* 7.3 (SCLC)	*τ_s_* = 18 ± 6 *τ_b_* = 570 ± 69 (TA)	1.8–10.0	For hole: (1.4 ± 0.2) × 10^10^ (SCLC)	—
MAPbI_3_ (TSSG) ^(3)^	For hole: 105 *±* 35 (Hall effect); 164 *±* 25 (SCLC) For electron: 24.8 *±* 4.1 (SCLC); 24.0 *±* 6.8 (ToF)	Under 1 sun: 8.2 × 10^4^ (TPV) ^(8)^ 9.5 × 10^4^ (IS) ^(9)^ Under 0.1 sun: 2.3 × 10^5^ (TPV) 2.0 × 10^5^ (IS)	For hole: 175 ± 25	For hole: 3.6 × 10^10^ For electron: 4.5 × 10^10^ (SCLC)	For hole: (9 *±* 2) × 10^9^ (Hall effect)
MAPbI_3_	68 (SCLC)	*τ_s_* = 94 *τ_b_* = 493 (TRPL)	4.0	—	—
a-FAPbI_3_ (ITC)	4.4 (SCLC) 1.07 ± 0.25 (ToF)	*τ_s_* = 32 *τ_b_* = 484 (TRPL)	0.5–2.2	6.2 × 10^11^ (SCLC)	1.5 × 10^11^
FAPbI_3_	41 (SCLC)	*τ_s_* = 79 *τ_b_* = 1393 (TRPL)	2.9	—	—

^(1)^ ITC—inverse temperature crystallization, ^(2)^ ACS—vapor-assisted crystallization, ^(3)^ TSSG—top-seeded solution growth, ^(4)^ SCLC—space charge limited current, ^(5)^ ToF—time of flight, ^(6)^ TA—transient absorption, ^(7)^ TRPL—time-resolved photoluminescence, ^(8)^ TPV—transient photovoltage, ^(9)^ IS—impedance spectroscopy.

**Table 3:** The comparison of PC and PV detectors.

Parametr	PC Detector	PV Detector	Schematic Figures
Gain (*g*)	$g=\frac{\tau }{\tau_{t}}=\frac{\tau \mu_{e}V_{b}}{l^{2}}$ ^(1)^	g=1 (for APD >> 1) ^(2)^	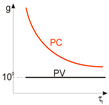
Responsivity (*R*)	$R_{v}=\frac{V_{s}}{P_{\lambda }}=\frac{\eta }{lwt}\frac{\lambda \tau }{hc}\frac{V_{b}}{n_{o}}$ ^(3)^	$R_{i}=\frac{I_{s}}{P_{\lambda }}=\frac{q}{hc}$ ^(4)^	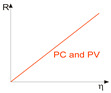
Noise	$\bar{i_{gr}^{2}}=\frac{4qIg∆f}{1+\omega^{2}\tau^{2}}$ ^(5)^	$\bar{i_{sh}^{2}}=2qI∆f=2q\left(I_{0}e^{qV/kT}+I_{0}\right)∆f$ ^(6)^	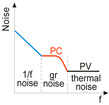
Noise equivalent power (*NEP*)	$NEP=\frac{v_{n}}{R_{v}}$ ^(7)^	$NEP=\frac{i_{n}}{R_{i}}$ ^(8)^	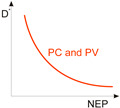
Detectivity (D*)	$D^{*}=\frac{{\left(A∆f\right)}^{1/2}}{NEP}=\frac{R_{v}{\left(lw∆f\right)}^{1/2}}{{\left(\bar{v_{J}^{2}}+\bar{v_{gr}^{2}}\right)}^{1/2}}$ ^(9)^	$D^{*}=\frac{{\left(A∆f\right)}^{1/2}}{NEP}=\frac{\eta \lambda q}{hc}{\left(\frac{4kT}{R_{0}A}+2q^{2}\eta \phi_{b}\right)}^{-1/2}$ ^(10)^	
BLIP detectivity	$D_{BLIP}^{*}=\frac{\lambda }{2hc}{\left(\frac{\eta }{\phi_{B}}\right)}^{1/2}$	$D_{BLIP}^{*}=\frac{\lambda }{hc}{\left(\frac{\eta }{{2\phi }_{B}}\right)}^{1/2}$	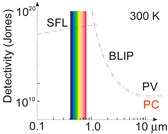
SFL detectivity	$D_{SFL}^{*}=\frac{\eta \lambda }{2^{3/2}hc}{\left(\frac{A}{\Delta f}\right)}^{1/2}$	DSFL*=ηλ23/2hcAΔf1/2	
Linear dynamic range (LDR)	$R_{v}\propto P_{\lambda }^{-(1-\alpha )}$, where *a* ≈ 1	$I_{ph}\propto P_{\lambda }^{\alpha }$, where *a* ≈ 1	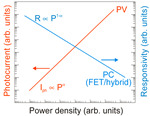

**^(1)^** *τ*—carrier lifetime, *τ_t_*—carrier transit time, *l*—carrier transit length, *m_e_*—carrier mobility, *V_b_*—voltage; **^(2)^** APD—avalanche photodetector; **^(3)^** *V_s_*—the output rms voltage, *P_λ_*—incident irradiance power, *η*—quantum efficiency (QE), *w*—detector width, *t*—detector thickness, *λ*—light wavelength, *h*—Planck constant, *c*—light velocity, *n*_0_—majority carrier concentration in the n-type material; **^(4)^** *I_s_*—output rms current, **^(5)^** *q*—electron charge, $\bar{i}$—average current, *g*—PC gain; Δ*f*—detector’s operating bandwidth; **^(6)^** $\bar{i_{sh}}$—shot noise, *I*—total current; *V*—voltage; *k*—Boltzmann constant, *T*—operating temperature; **^(7)^** *v_n_*—rms noise voltage, *R_v_*—voltage responsivity; **^(8)^** *i_n_*—rms noise current, *R_i_*—current responsivity; **^(9)^** *A*—detector’s photosensitive area, $\bar{v_{J}}$—Johnson noise voltage, $\bar{v_{gr}}$—generation-recombination noise voltage; **^(10)^** *R*_0_*A*—zero-bias resistance and photosensitive area product; *ϕ_b_*—background radiation flux density.

**Table 4:** Perovskite photodetectors—device/materials/performance.

Device/Materials	Morphology	Spectral Region [nm]	*R* [A/W] @ *V_bias_* [V], (*l* [nm], *P* [W/cm^2^])	D* [Jones] @ *V_bias_* [V] (*l* [nm], *P* [mW/cm^2^])	LDR [dB]	Rise Time/Fall Time	Ref.
Photodiodes							
PD/MAPbI_3−x_Cl_x_	PF	300–800	-	8 × 10^13^ @ −0.1 (550, 1)	>100	180 ns/160 ns	[11]
PD/MAPbI_3_	PF	300–800	-	~3 × 10^12^ @ 0 (700)	~170	1.7 μs/1.0 μs	[11]
PD/MAPbCl_3_	SCR	365	~0.05 @ −15 (365, 1)	1.2 × 10^10^	-	24 ms/62 ms	[11]
PD/MAPbBr_3_	SCR	~555–585	-	2 × 10^10^ @ 4 (570)	-	~1.6 ms	[11]
Photoconductors							
PC/MAPbBr_3_	SCR	380–600	>4000 @ 5 (525)	>3 × 10^13^	-	~25 μs/25 μs	[39]
PC/MAPb(Br_x_I_1−x_)_3_	SCR	405–710	2.36 A/W @ 2 (460)	1.15 × 10^12^	-	3.4 mqs/3.6 ms	[11]
PC/MAPbI_3_	NWs	400-750	4.95 @ 1 (530, 2 n)	2 × 10^13^	-	<0.1 ms	[11]
PC/Cs_x_(MA)_1−x_PbI_3_	NW	530	23 @ 5 (530, 4.5 m)	2.5 × 10^11^	-	-	[11]
PC/MAPbI_3_	NW	370–780	12500 @ 5 (550, 42 m)	1.73 × 10^11^	114	0.34 μs/0.42 μs	[11]
PC/CsPbBr_3_+Au nanoparticles	NC	300–550	~0.01 @ 2 (520)	4.56 × 10^8^	-	0.2 ms/1.2 ms	[11]
PC/MAPbI_3_	2D/quasi-2D	405	22 @ 1 (405)	-	-	20 ms/40 ms	[11]
PC/CsPbBr_3_	2D/quasi-2D	300–550	0.64 @ 10 (517)	-	-	19 μs/25 μs	[11]
PC/CsPbBr_3_	NC	450	6.4 × 10^4^ @ 3	≈10^13^		0.5 ms/1.6 ms	[40]
(PEA)_2_PbI_4_ 0.6 μm thick 2D plates	NC	460	98.2 @ 4	1.6 × 10^15^		64 ms/52 μs	[41]
Field effect phototransistors							
FET/MAPbI_3−x_Cl_x_	NC	300–800	5.6 × 10^8^	2.8 × 10^16^	92	20 μs/0.445 ms	[11]
FET/CsPbBr_3−x_I_x_-graphene	NC	400–700	8.2 × 10^8^ @ (405, 0.07 m)	~10^16^ @ (405, 0.07)	-	0.81 s/3.65 s	[29]
FET/(C_4_H_9_NH_3_)_2_PbBr_4_-graphene	2D/quasi-2D	470	~2100 @ 0.5 (470, 1 m)	-	-	-	[11]
FET/FAPbI_3_QD/VAGA	QD	1550	2.2 × 10^7^	5.6 × 10^15^	-	46 ms/46 ms	[32]
FET/CNT/CsPbBr_3_QD	QD	405	5.1 × 10^7^ @ (405, 10 n)	2 × 10^16^		3 ms	[43]
FET/PDOT:PSS/CH_3_NH_3_PbI_3−x_Cl_x_		598	1.9 × 10^9^ @ (*V_D_* = 0.5 V)	1.4 × 10^14^		57.5 s	[42]
FET(ThMA)_2_(MA)_n−1_Pb_n_I_3n+1_ (n = 3)	NW	530	1.1 × 10^4^ @ 5	9.1 × 10^15^ @ 5		36.2–31.5 μs	[44]
FET/(BA)_2_(MA)_n−1_Pb_n_I3_n+1_ (n = 4)	NW	530	1.5 × 10^4^ @ 5	7 × 10^15^ @ 5		27.6–24.5 μs	[45]
FET/s-CNT/(PEA)_2_SnI_4_	NC	532	6.3 × 10^4^ (*V_DS_* = 10 V, *V_G_* = −40 V)	1.12 × 10^17^		825 ms/440 ms	[46]
FET/FAPbBr_3_/-graphene	QD	650	1.15 × 10^5^	‒		58 ms/60 ms	[47]
FET/PQDs/MoS_2_MvdWH	QD	670	7.7 × 10^4^	5.6 × 10^11^		590 ms/320 ms	[48]

PD—photodiode, PC—photoconductor, FET—field effect transistor, PF—polycrystalline, SCR—single crystal, NW—nanowires, NC—nanocrystals, QD—quantum dots, *R*—responsivity, D*—detectivity, *P*—Intensity.

**Table 5:** The key polymer parameters implemented into flexible photodetectors.

Material	Light Transmission (%)	Dimensional Stability	Temperature Tolerance (°C)	Solvent Resistance	Elastic Modulus (MPa)
Polyethylenenaphthalate (PEN)	87.0	Well	120	Well	6000
Polyethylene terephthalate (PETP	90.4	Well	79	Well	4000
Polyvinylidene fluoride (PCDF)	25–30	Well	150	Well	1400
Polimide (PI)	30–60	Well	280	Well	500
Polydimethylsiloxane (PDMS)	93	Fair	260	Fair	150
Poly(methyl methacrylate (PMMA)	92	Fair	100	Fair	6500

## Data Availability

Dataset available on request from the authors.
